# Visceral Adipose Tissue Phospholipid Signature of Insulin Sensitivity and Obesity

**DOI:** 10.1021/acs.jproteome.0c00918

**Published:** 2021-03-24

**Authors:** Magalí Palau-Rodriguez, Anna Marco-Ramell, Patricia Casas-Agustench, Sara Tulipani, Antonio Miñarro, Alex Sanchez-Pla, Mora Murri, Francisco J. Tinahones, Cristina Andres-Lacueva

**Affiliations:** † Biomarkers and Nutrimetabolomics Laboratory, Department of Nutrition, Food Sciences and Gastronomy, XIA, INSA, Faculty of Pharmacy and Food Sciences, University of Barcelona, Barcelona 08028, Spain; ‡ CIBER Fragilidad y Envejecimiento Saludable (CIBERfes), Instituto de Salud Carlos III, Madrid 28029, Spain; § Department of Endocrinology and Nutrition, Instituto de Investigación Biomédica de Malaga (IBIMA), Virgen de la Victoria University Hospital,, Málaga University, Malaga 29010, Spain; ∥ Genetics, Microbiology and Statistics Department, Biology Faculty, University of Barcelona, Barcelona 08028, Spain; ⊥ CIBER Fisiopatología de la Obesidad y Nutrición (CIBERobn), Instituto de Salud Carlos III, Madrid 28029, Spain

**Keywords:** discordant phenotypes, insulin resistance, lipid remodeling, metabotype, metabolomics, phospholipids, obesity, diabetes, biomarker

## Abstract

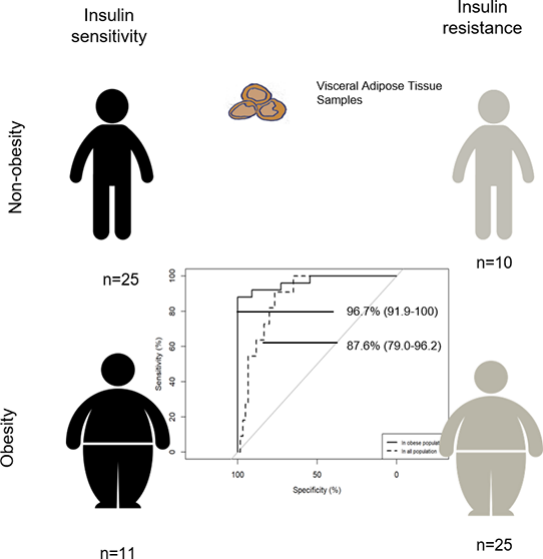

Alterations in visceral adipose tissue (VAT) are closely linked to cardiometabolic abnormalities. The aim of this work is to define a metabolic signature in VAT of insulin resistance (IR) dependent on, and independent of, obesity. An untargeted UPLC-Q-Exactive metabolomic approach was carried out on the VAT of obese insulin-sensitive (IS) and insulin-resistant subjects (*N* = 11 and *N* = 25, respectively) and nonobese IS and IR subjects (*N* = 25 and *N* = 10, respectively). The VAT metabolome in obesity was defined among other things by changes in the metabolism of lipids, nucleotides, carbohydrates, and amino acids, whereas when combined with high IR, it affected the metabolism of 18 carbon fatty acyl-containing phospholipid species. A multimetabolite model created by glycerophosphatidylinositol (18:0); glycerophosphatidylethanolamine (18:2); glycerophosphatidylserine (18:0); and glycerophosphatidylcholine (18:0/18:1), (18:2/18:2), and (18:2/18:3) exhibited a highly predictive performance to identify the metabotype of “insulin-sensitive obesity” among obese individuals [area under the curve (AUC) 96.7% (91.9–100)] and within the entire study population [AUC 87.6% (79.0–96.2)]. We demonstrated that IR has a unique and shared metabolic signature dependent on, and independent of, obesity. For it to be used in clinical practice, these findings need to be validated in a more accessible sample, such as blood.

## Introduction

The prevalence of obesity is increasing dramatically, making it one of the biggest public health challenges across the world. Other comorbidities, including cardiovascular complications, type 2 diabetes, and insulin resistance (IR), usually accompany obesity, but when obesity and IR occur simultaneously, it hampers the study of the physiopathology associated with obesity or IR itself.

Adipose tissue, which is a metabolically dynamic organ, is the primary storage site for excess energy, while at the same time being an endocrine organ that synthesizes numerous biologically active compounds that regulate metabolic homeostasis. It has been stated that obesity is the result of excessive growth of adipose tissue depots. When the size, expandability,^1^ and functionality^2^ of the adipose tissue depots vary significantly it results in a chronic state of “low-grade” inflammation that is related to a diverse risk of developing comorbidities linked to obesity.^3^ Furthermore, the risk of developing metabolic alterations may also be influenced by changes in the secretion of these active compounds, including adiponectin, leptin, and proinflammatory molecules.^1,4^ The existence of discordant phenotypes, as well as obese subjects with high insulin sensitivity and nonobese subjects with high insulin resistance, implies that the composition of adipose tissue, rather than the amount of fat, may play a key role in studying the development of insulin resistance dependent on, and independent of, obesity. Because of the nature of this sample, though, and the difficulty involved in getting hold of large amounts, few studies have analyzed the composition of visceral adipose tissue (VAT), particularly in nonobese subjects.

It is thought that visceral adipose tissue (VAT) is different, both functionally and metabolically, from adipose tissue types, including subcutaneous adipose tissue (SAT). SAT is less metabolically active than VAT and is described as an active endocrine organ whose complex roles go beyond energy storage.^5^ Changes in VAT are closely related to cardiometabolic disorders.^6^ A global assessment of the metabolic status of the VAT of obese individuals with high insulin sensitivity (IS) or high IR using a metabolomic-driven approach will enable these metabolic phenotypes (metabotypes) to be profiled and allow potential markers of metabolic healthy obesity (MHO) to be discovered.

The aims of the present study were to (1) explore the VAT metabolome of obese and nonobese subjects with high and low IR and the associated pathways, (2) identify metabolomic differences between metabotypes, and (3) explore the potential of metabolites as promised candidate biomarkers of VAT in obese individuals with MHO or high IR. To achieve these aims, we adopted an untargeted metabolomic-driven approach on the VAT of human concordant and discordant phenotypes of obesity and high IR. Univariate and multivariate statistical analysis, regression analysis for variable selection, receiver operating characteristic (ROC) curves, and pathway enrichment analysis were employed to analyze the data. The comprehensive analysis of the metabolome of concordant and discordant phenotypes of obesity and high IR may open the way for potential metabolites as candidate biomarkers of visceral fat in obese subjects with MHO or high IR.

## Experimental Section

### Subjects and Study Design

A total of 71 adults, comprising 27 men and 44 women, were recruited from the Virgen de la Victoria University Hospital in Malaga, Spain. The study design and inclusion/exclusion criteria have previously been described in detail.^7^ In brief, patients suffering from either an acute or chronic disease, including type 2 diabetes, or who were on antihyperglycemic agents, insulin, or any potential lipid profile-altering drugs, were excluded.

Individuals were classified in this cross-sectional study according to their body mass index (BMI) into nonobese (BMI = 18.5–26.9 kg/m^2^) or morbidly obese (BMI > 40 kg/m^2^) subjects and according to their risk of developing type 2 diabetes based on fasting plasma glucose (FG) concentrations and homeostatic model assessment-IR (HOMA-IR) as follows: low IR or IS state (FG < 100 mg/dL and HOMA-IR < 2.5) or high IR state (FG levels 100–125 mg/dL or HOMA-IR > 3.4). The HOMA-IR cutoff was established experimentally by dividing the whole of the initial cohort into quartiles as previously described.^7^ Participants were categorized into the following four sex-matched phenotypic groups: (1) nonobese subjects with low IR or IS, referred to as the control group (*N* = 25), (2) obese subjects with IS (*N* = 11), (3) nonobese subjects with high IR (*N* = 10), and (4) obese subjects with high IR (*N* = 25), as previously described.^7^

Standardized techniques were employed to measure anthropometric values and parameters, as previously described.^7,8^ Laparoscopic surgery was carried out to obtain biopsies of VAT, which were then frozen at −80 °C until assayed. The local Ethics and Research Committee (Hospital Universitario Virgen de la Victoria, Málaga, Spain) approved the protocol, and written informed consent was obtained from all participants.

### Metabolomic Profiling

Sample preparation and analytical metabolomic analysis were carried out at Metabolon Inc. (Durham, NC). The automated MicroLab STAR system (Hamilton, Bonaduz, Switzerland) was used to prepare tissue extracts with methanol, as previously described.^9^

Two extracts were used for reverse-phase ultraperformance liquid chromatography–tandem mass spectrometry (RP-UPLC–MS/MS) with positive ion mode electrospray ionization (ESI+) in acidic conditions for hydrophilic and hydrophobic compounds and one extract with negative ionization (ESI−) in basic conditions. A fourth extract was analyzed by hydrophilic interaction chromatography (HILIC)/UPLC-MS/MS ESI–. Aliquotes were analyzed using an ACQUITY UPLC system (Waters, Milford, MA), coupled with a Q Exactive mass spectrometer and an Orbitrap mass analyzer (both from Thermo Scientific, Waltham, MA). The UPLC system was equipped with a UPLC C18 BEH (2.1 × 100 mm^2^, 1.7 μm) or UPLC BEH Amide (2.1 × 150 mm^2^, 1.7 μm) column (Waters). The Q Exactive system was interfaced with a heated ESI (HESI-II) source and an Orbitrap operated at 35 000 mass resolutions. The mass range was 70–1000 *m*/*z*, and the MS analysis alternated between MS and data-dependent MSn scans using dynamic exclusion. More information was detailed previously.^9^ To determine instrument variability, the relative standard deviation (RSD) was calculated for the internal standards that were added to each sample prior to injection into the MS. The RSD for all endogenous metabolites present in all of the samples was calculated to determine the total process variability using replicates of pooled human samples injected periodically during the platform run. The median RSD of the analytical platform instrumentation was 3%, while the median RSD of the overall process variability was 7%. These values indicated acceptable levels of variability for both instrument and overall process variability.

The peak area was used to quantify peaks. Compounds were identified by comparing them to a Metabolon library that contains more than 4000 purified standard entries. Three criteria were used to identify metabolites: the retention time/index (RI), the mass-to-charge ratio (*m*/*z*)′, and chromatographic data (including MS/MS spectral data) on all molecules present in the library, considering an accurate mass match to the library of ±10 ppm.^10^

### Statistical Analysis

Data analysis was conducted in R (v.3.4). First, to remove ion compounds with more than 80% of values missing in all groups, the data set was filtered. Next, data were logarithmically transformed and Pareto-scaled and the gender, age, and drug consumption were included as covariables in the analysis of variance (ANOVA) model and for multivariate analysis using the residual matrix of their effects. In the latter case, data were first imputed with the *k*-nearest neighbor algorithm (*k* = 5).^11^

To describe clinical and metabolic parameters, univariate analysis was carried out. An ANOVA for unbalanced groups was conducted to evaluate the effects of obesity and high IR and to compare clinical and metabolic parameters between groups. Based on the Benjamini–Hochberg procedure, all *p*-values were also corrected for multiple testing by the false discovery rate (FDR)^12^ and the only metabolites considered significant were those with adjusted *p*-values < 0.05.

Random forest (RF) modeling within an in-house-developed repeated double cross-validation (rdCV) was employed to find the most discriminative metabolites between groups.^13^ The following parameters were set: number of repetitions = 20, metabolites in the outer loop = 5, varRatio = 0.8, and number of permutations = 200. The rdCV minimizes statistical overfitting, improves the accuracy of modeling, and reduces misclassifications. More information on this procedure can be found in refs (14, 15). The stability of the model was evaluated through the misclassification rate (<20%) and the fitness of randomly permuted classifications (*p*-value < 0.05).

### Models of Classification of “IS Obesity” Metabotype

Variable selection was carried out with the least absolute shrinkage and selection operator (LASSO) logistic regression utilizing a leave-one-out cross-validation with metabolites with a *p*-value of <0.05 and an adjusted *p*-value of <0.25. The LASSO method is a multivariate regression model that penalizes those metabolites that do not contribute to the model. Therefore, the most predictive metabolites are selected for the model, while the coefficients of the remaining metabolites are reduced to zero.^16^ A new parameter is created using the coefficients of the most predictive metabolites, the multimetabolite biomarker. The global performance of this biomarker model and its components was assessed by receiver operating characteristic (ROC) curves, namely, area under the curve (AUC) value, confidence intervals (CIs 95%), sensitivity, and specificity. The performance of the multimetabolite biomarker was also evaluated in the nonobese population.

### Enrichment and Correlation Analysis

A hypergeometric test was used to conduct enrichment analysis. For estimating the associations among the selected metabolites and clinical variables, Spearman correlation coefficients were calculated, and they were represented as a hierarchically clustered correlation matrix with average distance.

The LASSO regression and ROC curves were performed using the *glmnet* and *pROC* packages, respectively. The *Hmisc* and *ggplot* packages were used, respectively, for the analysis of correlations and the creation of a heatmap.

## Results

### Clinical Data

Obese subjects presented increased adiposity indicators, including BMI, weight, and hip and waist circumferences, as well as raised levels of liver damage markers and uric acid, and decreased levels of cholesterol associated with high-density lipoprotein (HDL) particles than normal-weight individuals. Individuals with high IR showed increased concentrations of IR indicators, including fasting glucose and insulin and HOMA-IR, and raised levels of triglycerides and cholesterol associated with low-density lipoprotein cholesterol (LDL) particles and uric acid. No significant changes were noted in the interaction of obesity × high IR. Concordant and discordant phenotypes of obesity (IR obesity versus high IS obesity) showed differences in IR markers, as expected, whereas phenotypes of high IR (high IR nonobesity versus high IR obesity) did not in adiposity and liver damage markers (Table 1).

**Table 1:** Anthropometric and Clinical Parameters of the Subjects of the Study^a^

							adjusted *p*-value			
	non-OB IS	non-OB IR	OB IS	OB IR	OB	IR	OB × IR	OB IS versus OB IR	non-OB IR versus OB IR	non-OB IS versus non-OB IR
gender	M = 7, F = 18	M = 3, F = 7	M = 3, F = 8	M = 14, F = 11	n.s.	n.s.	n.s.	n.s.	n.s.	n.s.
age [years]	47.56 ± 13.66	54.90 ± 13.62	40.36 ± 10.64	42.84 ± 8.99	0.004	n.s.	n.s.	n.s.	0.003	n.s.
weight [kg]	64.56 ± 8.64	66.10 ± 5.63	125.97 ± 15.00	154.80 ± 31.20	5.92 × 10^–24^	0.040	n.s.	n.s.	1.08 × 10^–8^	n.s.
BMI [kg/cm^2^]	23.75 ± 2.12	25.30 ± 1.64	46.14 ± 4.58	53.63 ± 9.05	5.38 × 10^–26^	0.009	n.s.	n.s.	1.08 × 10^–8^	n.s.
waist [cm]	81.88 ± 7.93	92.70 ± 3.65	128.00 ± 16.18	146.62 ± 21.73	1.69 × 10^–18^	0.004	n.s.	n.s.	2.34 × 10^–6^	0.038
hip [cm]	94.88 ± 8.26	101.30 ± 3.66	142.40 ± 11.07	151.25 ± 17.80	1.78 × 10^–18^	0.031	n.s.	n.s.	2.78 × 10^–6^	n.s.
waist/hip [ratio]	0.81 ± 0.04	0.90 ± 0.67	0.87 ± 0.11	0.97 ± 0.11	n.s.	n.s.	n.s.	n.s.	n.s.	n.s.
fasting glucose [mmol/L]	89.96 ± 7.59	115.50 ± 14.93	88.82 ± 4.58	115.68 ± 11.63	n.s.	2.67 × 10^–13^	n.s.	1.15 × 10^–7^	n.s.	4.34 × 10^–5^
fasting insulin [μU/mL]	5.33 ± 2.04	12.36 ± 4.04	8.19 ± 2.16	24.81 ± 13.25	0.003	1.03 × 10^–10^	n.s.	2.89 × 10^–6^	n.s.	9.13 × 10^–4^
HbA1c [%] (mmol/mol)	5.32 ± 0.40 (34.59 ± 4.36)	6.14 ± 0.61 (43.59 ± 9.24)	5.63 ± 0.10 (37.96 ± 1.05)	5.69 ± 0.47 (38.68 ±5.11)	n.s.	n.s.	n.s.	n.s.	n.s.	0.002
HOMA-IR [index]	1.18 ± 0.47	3.46 ± 0.99	1.80 ± 0.51	7.10 ± 3.81	0.004	5.75 × 10^–14^	n.s.	1.15 × 10^–7^	n.s.	4.34 × 10^–5^
systolic pressure [mm Hg]	115.42 ± 13.39	133.70 ± 27.61	131.50 ± 22.55	135.61 ± 18.38	n.s.	n.s.	n.s.	n.s.	n.s.	n.s.
diastolic pressure [mm Hg]	71.88 ± 11.54	79.20 ± 8.61	82.00 ± 10.73	84.33 ± 13.43	n.s.	n.s.	n.s.	n.s.	n.s.	n.s.
total cholesterol [mmol/L]	179.20 ± 25.26	241.60 ± 42.61	190.18 ± 50.15	201.76 ± 34.53	n.s.	0.003	n.s.	n.s.	n.s.	0.004
HDL [mmol/L]	55.80 ± 10.59	55.60 ± 17.61	45.73 ± 15.43	39.92 ± 12.95	0.022	n.s.	n.s.	n.s.	n.s.	n.s.
LDL [mmol/L]	105.74 ± 24.40	153.04 ± 41.62	107.51 ± 45.71	134.50 ± 28.57	n.s.	8.28 × 10^–4^	n.s.	n.s.	n.s.	0.018
triglycerides [mmol/L]	82.36 ± 39.52	166.00 ± 78.62	121.64 ± 112.87	144.47 ± 48.28	n.s.	0.003	n.s.	n.s.	n.s.	0.010
C-reactive protein [mg/L]	2.98 ± 0.77	5.95 ± 2.62	13.80 ± 12.51	8.87 ± 6.24	n.s.	n.s.	n.s.	n.s.	n.s.	0.010
GOT [U/L]	14.60 ± 6.24	14.60 ± 9.62	21.91 ± 8.87	24.64 ± 11.84	1.96 × 10^–4^	n.s.	n.s.	n.s.	0.018	n.s.
GPT [U/L]	27.48 ± 9.29	32.50 ± 14.62	38.91 ± 16.49	54.20 ± 19.26	2.32 × 10^–4^	n.s.	n.s.	n.s.	0.018	n.s.
GGT [U/L]	23.64 ± 14.86	37.35 ± 47.62	26.27 ± 17.86	53.88 ± 50.74	0.006	n.s.	n.s.	n.s.	0.003	n.s.
uric acid [mmol/L]	3.74 ± 0.84	4.76 ± 1.62	5.04 ± 0.78	6.49 ± 1.46	6.01 × 10^–5^	0.003	n.s.	n.s.	n.s.	0.045
creatinine [mmol/L]	0.77 ± 0.20	0.77 ± 0.62	0.74 ± 0.17	0.84 ± 0.18	n.s.	n.s.	n.s.	n.s.	n.s.	n.s.
urea [mg/dL]	30.05 ± 7.95	35.00 ± 7.62	27.90 ± 7.82	32.68 ± 12.13	n.s.	n.s.	n.s.	n.s.	n.s.	n.s.

a Values are shown as mean ± SD unless otherwise indicated. *p*-Values were calculated using the ANOVA model and t-test after log-transformed variables were appropriated and corrected by the false discovery rate. Abbreviations: BMI, body mass index; LDL, low-density lipoproteins cholesterol; HDL, high-density lipoproteins cholesterol; GOT, aspartate transaminase; GPT, alanine transaminase; GGT, γ-glutamyl transferase; F, female; HbA1c, glycated hemoglobin A1c; HOMA-IR, insulin resistance calculated by homeostatic model assessment; IR, high insulin resistance; IS, insulin sensitivity; M, male; n.s., not significant; OB, obesity; SD, standard deviation.

### VAT’s Metabolic Profiling Obesity

Four hundred and twenty-two different metabolites were identified among all of the groups of the study. A total of 118 various metabolites were revealed through the use of univariate statistics in the VAT metabolome of obese compared to nonobese subjects (adjusted *p*-value of the interaction obesity × high IR > 0.05) (Table S1). Changes in these metabolites reflected changes in the following six metabolic pathways: (1) metabolism of leucine, isoleucine, and valine; (2) tricarboxylic acid (TCA) cycle; (3) metabolism of glutathione; (4) glycolysis and gluconeogenesis; (5) metabolism of glycerolipids; and (6) metabolism of glycine, serine, and threonine (Table S2).

**Figure 1:**
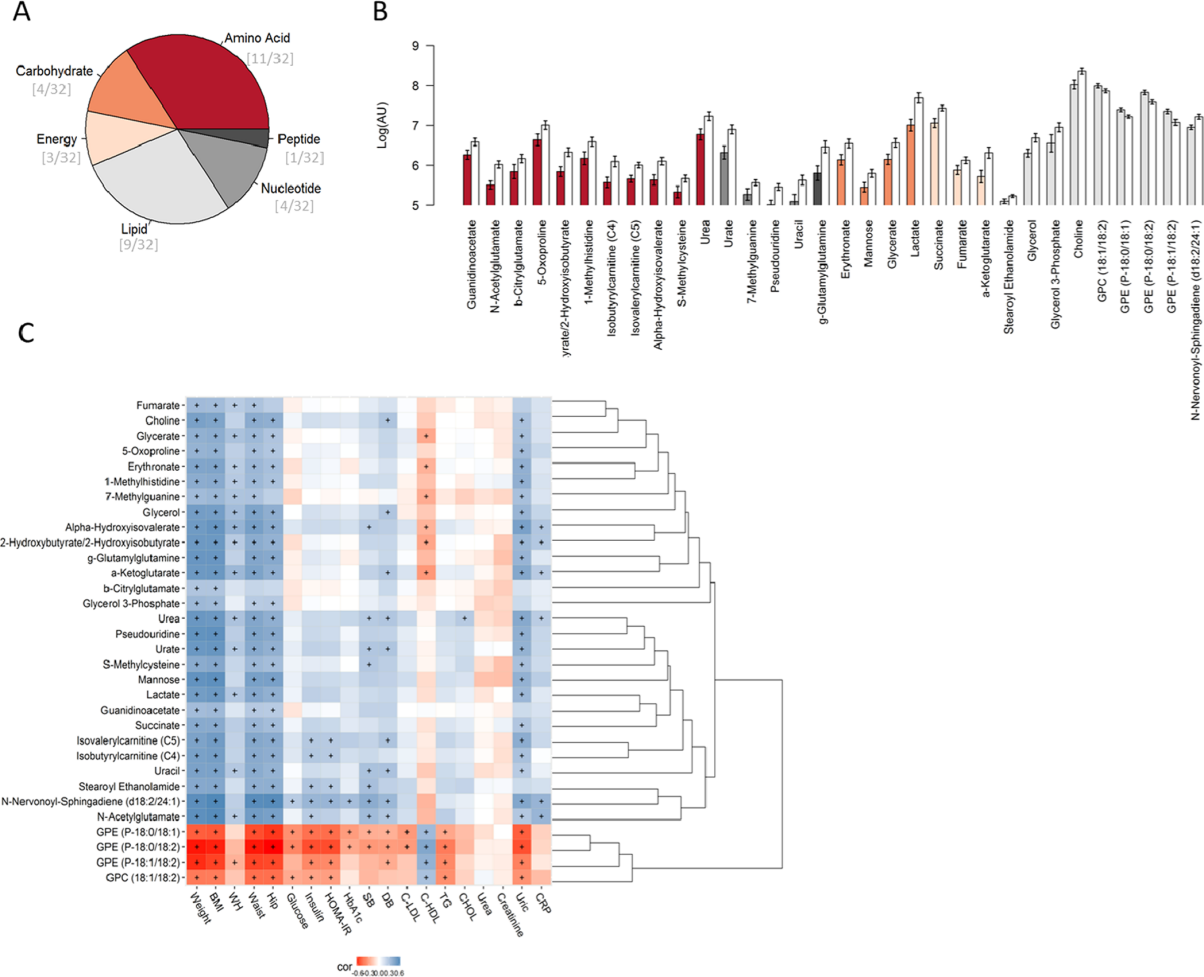
Selected metabolites from the comparison between obese and nonobese subjects by random forest. (A) Summary of the chemical classes of the selected metabolites. (B) Mean and standard error of the logarithmic transformation of the levels of the discriminant metabolites in the obese subjects (white bars) and those of normal weight (colored and sorted according to the class of the metabolites). (C) Hierarchical clustered Spearman correlation matrix of the selected metabolites and anthropometric and clinical parameters by random forest analysis of obese and nonobese subjects. Adjusted *p*-values with the significant threshold set at <0.05 are marked with +. Positive correlations are in blue, and negative correlations are in red.

The random forest model picked 32 of these metabolites that distinguished between obese and normal-weight subjects (Figure 1A,B and Table S3) with a 15% misclassification and *p*-value < 0.001. The ten most discriminative metabolites among these metabolites were urate, lactate, N-acetylglutamate, urea, 2-hydroxy(iso)butyrate, succinate, two plasmalogens glycerophosphaethanolamine (GPE), α-hydroxyisovalerate, and γ-glutamylglutamine.

The correlation analysis revealed negative correlations of glycerophosphatidylcholines and plasmalogen GPE with clinical data except for HDL particles. Positive correlations were noted between all of the other 28 metabolites and weight-related parameters. Furthermore, there were also positive correlations between N-nervonoyl-sphingadiene (d18:2/24:1), N-acetylglutamate, steroyl ethanolamide, isobutyrylcarnitine and isovalerylcarnitine (carnitines C4 and C5, respectively), and glycemic parameters. Blood pressure correlated positively with choline, glycerol, α-hydroxyisovalerate, α-ketoglutarate, urea, urate, S-methylcysteine, carinitine C5, uracil, stearoyl ethanolamine, N-nervonoyl-sphingadiene (d18:2/24:1), and N-acetylglutamate, while HDL particles were negatively correlated with glycerate, erythronate, 7-methylguanine, α-hydroxyisovalerate, 2-hydroxyisobutyrate, and α-ketoglutarate (Figure 1C).

### VAT’s Metabolic Profiling in High IR

There were no differences in VAT metabolome in subjects with high IR when they were compared with subjects with IS. The effect of high IR in the metabolome depending on the obesity variable was neither observed. Higher levels of plasmalogen GPE (P-18:0/18:2) were observed in subjects with IS, *p*-value = 0.001 (adjusted *p*-value = 0.077). Moreover, the RF analysis was unable to distinguish clearly between subjects with IS and those with high IR.

### VAT Metabolome of Discordant Phenotypes: Nonobesity with High IR and Obesity with IS

The metabolites that distinguished between high IR phenotypes (nonobese versus obese subjects with high IR) were the same as those observed in the comparison between nonobesity and obesity (Table S1). No differences were noted between nonobese phenotypes (nonobese versus obese subjects with high IR).

**Figure 2:**
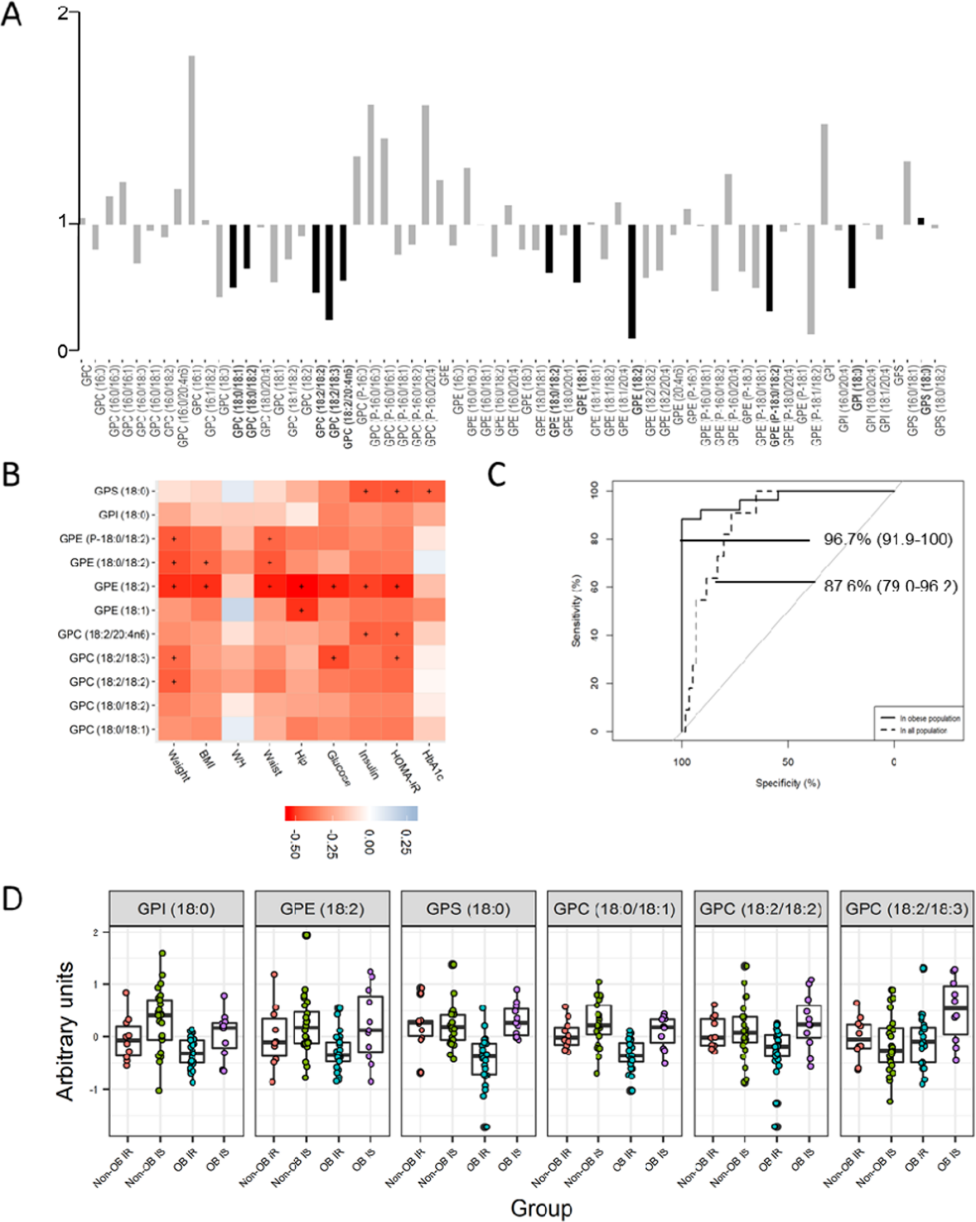
VAT metabolome of the discordant phenotype of obesity. (A) Fold changes in the levels of lipid species between obese subjects with high insulin resistance and nonobese subjects with insulin sensitivity. Lipids significantly different between groups were marked in a dark color and with bold text (adjusted *p-*value < 0.25). (B) Spearman correlation matrix of the selected lipids with clinical variables. Adjusted *p*-values with a cutoff at <0.05 are marked with +. Positive correlations are in blue, and negative correlations are in red. (C) ROC curves (AUC%, CI 95%) of the multimetabolite biomarker model to identify the IS obesity metabotype among the obese population (IS and high IR) or all of the subjects of the study (normal weight and obesity and IS or high IR). The model was formed by GPE 18:2, GPI 18:0, GPS 18:0, GPC aa 36:1, GPC aa 36:4, and GPC aa 36:5, selected by the LASSO method. (D) Boxplot of the levels of the individual metabolites of the multimetabolite biomarker after logarithmic transformation and Pareto scaling. Abbreviations: AUC, area under the curve; CI, confidence interval; GPC, glycerophosphatidylcholine; GPE, glycerophosphatidylethanolamine; GPI, glycerophosphatidylinositol; PS, glycerophosphatidylserine.

Differences were only revealed in the levels of the metabolite lysolipid GPE (18:2) between IS obesity and “high IR obesity”. To be specific, the levels of this lipid species were lower in the high IR obesity metabotype. When, for descriptive purposes, we set the adjusted *p*-value to 0.25, lower levels of other phospholipids containing fatty acyl groups of 18 carbons (C18) were also identified in the high IR obesity metabotype. The phospholipids concerned were glycerophosphatidylinositol (GPI) (18:0), glycerophosphatidylserine (GPS) (18:0), lysolipids GPE (18:1), GPE (18:0/18:1), GPE (18:0/18:2), GPC (18:0/18:2), GPC (18:2/18:2), GPC (18:2/18:3), GPC (18:2/20:4n6), and plasmalogen GPE (P-18:0/18:2) (Figure 2A).

It can be seen in Figure 2B that these species correlated negatively with weight parameters. It is also worth noting that there were statistically significant correlations between GPE (18:2) and weight, waist, and hip circumferences, as well as fasting glucose, insulin, and HOMA-IR.

The classification model of the IS obesity metabotype was obtained using regression analysis based on the LASSO method. This method was employed for choosing those metabolites that explained more clearly the differences between the IS obesity and high IR obesity metabotype. GPE (18:2) demonstrated a very good ability to detect the IS obesity metabotype when analyzing the subset with obese subjects [AUC 89.1% (78.8–99.4)] and the whole study population, comprising both nonobese and obese subjects [AUC 71.8% (56.5–87.2)] (Table 2 and Figure 2C). However, when this lysolipid was combined with the C18-containing phospholipids GPI (18:0), GPS (18:0), GPC (18:0/18:1), GPC (18:2/18:2), and GPC (18:2/18:3) (Figure 2D), this metabolite panel’s discriminative ability rose, yielding values of AUC 96.7% (91.9–100) in the subset of obese subjects and AUC 87.6% (79.0–96.2) in the whole study population (Table 2 and Figures 2C and S1).

**Table 2:** ROC Curve Parameters of the Combined Multimetabolite Biomarker Model for Detecting Subjects with Obesity and IS and of the Individual Metabolites That Are Part of This Model^a^

detection of OB IS	sensitivity (%)	specificity (%)	AUC (95% CI)
Only in Obese Population			
combined multimetabolite model	88.0	100	96.7 (91.9–100)
GPE 18:2	76.0	90.9	89.1 (78.8–99.4)
GPI 18:0	76.0	81.8	77.5 (56.7–98.2)
GPS 18:0	84.0	72.7	78.8 (61.1–96.8)
GPC aa 18:0/18:1 (GPC aa 36:1)	88.0	63.6	79.3 (61.0–97.6)
GPC aa 18:2/18:2 (GPC aa 36:4)	72.0	72.7	69.5 (48.7–90.2)
GPC aa 18:2/18:3 (GPC aa 36:5)	72.0	72.7	71.6 (50.0–93.3)
In the Whole Study Population			
combined multimetabolite model	90.9	76.7	87.6 (79.0–96.2)
GPE 18:2	63.6	66.7	71.8 (56.5–87.2)
GPI 18:0	81.8	66.7	70.2 (50.3–90.0)
GPS 18:0	72.7	81.7	77.9 (61.9–93.9)
GPC aa 18:0/18:1 (GPC aa 36:1)	63.6	66.7	61.4 (43.3–79.5)
GPC aa 18:2/18:2 (GPC aa 36:4)	72.7	55.0	53.0 (35.1–70.9)
GPC aa 18:2/18:3 (GPC aa 36:5)	72.7	53.3	60.3 (39.1–81.5)

a Their predictive power was evaluated in the whole obese subset and in the whole study population including nonobese individuals. Metabolites are sorted alphabetically. Abbreviations: aa, diacyl; AUC, area under the curve; CI, confidence interval; GPC, glycerophosphatidylcholine; GPE, glycerophosphatidylethanolamine; GPI, glycerophosphatidylinositol; GPS, glycerophosphatidylserine.

The discriminative ability of the combined multimetabolite model between subjects with “IS nonobesity” in the nonobese population and in the whole study population was AUC 64.8% (44.19–85.4) and 49.9% (36.1–63.6), respectively, with a sensitivity and specificity of 70.0 and 68.0% and 52.0 and 58.7% in each case.

## Discussion

We provided a comprehensive VAT metabolic profiling of concordant/discordant phenotypes of obesity and high IR with a view to deepening the understanding of the complex relationship between obesity and high IR. To that end, we identified a VAT multimetabolite panel specific and sensitive to distinguishing obese patients with IS from those with high IR and also from the overall population. This panel demonstrated a modest ability to identify those subjects with normal weight and IS in the nonobese population and in the overall population.

First, the VAT metabolome of obese patients was extensively differentiated from that of normal-weight to overweight patients, in line with previous findings. Changes in the branched-chain amino acids (BCAAs) in obesity were revealed by the pathway analysis. Alterations in the metabolism of leucine, isoleucine, and valine were mirrored by changes in the metabolites, carnitines C4 and C5, coming from the metabolism of BCAA^17^ and α-hydroxyisovalerate. The levels of BCAA tend to be elevated in obese subjects, and increased levels of circulating BCAA are associated with imminent high IR or type 2 diabetes.^18^ In obese subjects, the impairment of the mitochondrial metabolism may lead to raised levels of C4 and C5 due to the reduction of fatty acid oxidation.^19^ In turn, mitochondrial dysfunction has been linked with high IR.^20^ However, there was no indication that the interaction of high IR × obesity was statistically significant in this study.

The lipidomic profiling of multiple populations and clinical cohorts has indicated that reduced levels of plasmalogens are linked with obesity, as well as with prediabetes and diabetes.^21^ Our findings showed that obese patients presented lower levels of phospholipids, specifically GPC and GPE plasmalogens, than those of normal weight, as previously observed.^22^ Plasmalogens are powerful antioxidants and upregulating them may decrease oxidative stress, ameliorate the lipid dysregulation that accompanies obesity, suppress inflammatory responses, and improve the high IR associated with metabolic diseases.

Comparisons between groups implied that the metabolism of phospholipids might also be modified by high IR in obese subjects but not in normal-weight patients. To be precise, GPE (18:2) and other phospholipid species also containing C18-fatty acyl groups, including GPE (18:1), GPI (18:0), GPS (18:0), GPC (18:0/18:1), GPC (18:2/18:2), and GPC (18:2/18:3), were lower in the high IR obesity metabotype. Regardless of adiposity, GPE (18:0/18:2) also exhibited lower levels in subjects with high IR than in those with IS. Phospholipids are formed by fatty acyl groups attached to their *sn*1 and *sn*2 of the glycerol backbone.^23^ They are first formed in the de novo pathway from glycerol-3-phosphate and then matured in the remodeling pathway. In this second step, the action of phospholipases (PL) A2 and phospholipid acyltransferases establish phospholipids’ asymmetry and high diversity.^24^ The phospholipid species GPC and GPE are the primary constituents of the plasma membrane, while GPI and GPS are less abundant in the cell.^25^ The enzyme PLA2 forms lysolipids from the cleavage of an acyl chain of phospholipids. Their role is a structural one, and they act as lipid mediators involved in cell signaling.^26^

Changes in arachidonyl-containing lipid species may be inconsistent with previous studies,^23,27^ as their levels were not significantly altered in obesity or high IR per se. Inflammation and oxidative stress are closely interconnected processes.^28^ The free radicals produced during the inflammatory reaction can also damage phospholipids, particularly plasmalogens, thereby reducing their levels.^29^ There is controversy over how the plasma membrane is affected by this lipid remodeling. While some report that the biophysical properties of the membrane are not altered,^27^ others suggest there are changes in membrane potential and permeability^30^ as well as altered receptor signaling.^26^ Obesity and related comorbidities lead to expansion, differentiation, and remodeling of adipocytes.^4^ Pietiläinen et al. studied the adipocyte remodeling in monozygotic twin pairs discordant for BMI. They found that the obese twins had higher proportions of palmitoleic (C16:1) and arachidonic (C20:4) acids in their adipose tissue and lower levels of both saturated fatty acids and linoleic (C18:2) and α-linoleic (C18:3) acids.^27^ Other studies found that arachidonyl-containing species increased during adipocyte differentiation, whereas linolenic-containing lipids decreased due to the raised activity level of the enzyme AT.^24^ Phospholipids with arachidonyl groups correlated positively with BMI and an increased risk of metabolic syndrome.^24^ On the other hand, we found no differences in levels of arachidonyl-containing species between obese and nonobese subjects but we did observe differences between obese subjects with high IR and those with IS. Engelmann et al. conducted a study on the erythrocyte plasma membrane in dyslipidaemia. These authors also found that under abnormal conditions the enzyme PLA2 is overexpressed and fatty acyl groups linked to GPE and GPC are transformed into arachidonyl-containing GPE and GPC.^23^ Arachidonyl acyl chains are further converted into proinflammatory markers, such as prostaglandins and eicosanoids, and intensify a proinflammatory response via PPARγ receptors.^23,24,27^ Pietiläinen et al. also pointed out that this proinflammatory environment induced by arachidonyl groups makes the adipocytes more vulnerable and prone to inflammatory responses and oxidation.^27^

These C18-fatty acyl-containing phospholipids demonstrated a high ability to discriminate between the IS obesity metabotype and the high IR obesity one, and also to identify it among the whole study population, including nonobese subjects. To be specific, GPE (18:2) was the metabolite that presented higher AUC and sensitivity and specificity rates. Furthermore, we performed a regression analysis to develop a multimetabolite biomarker model with a view to enhancing the prediction accuracy of the IS obesity metabotype. The resulting model consisted of a combination of GPE (18:2) with other 18 carbon-containing phospholipids: GPE (18:1), GPI (18:0), GPS (18:0), GPC (18:0/18:1), GPC (18:2/18:2) and GPC (18:2/18:3). This model demonstrated a very high ability to discriminate the IS obesity metabotype from high IR obesity and to differentiate it from all of the metabotypes of the study. However, the limited ability to distinguish subjects with IS nonobesity and a missing alternative biomarker in identifying this group within the whole population may suggest a different metabolic connection of IR in obese compared to nonobese subjects.

To the best of our knowledge, no studies report a biomarker based on metabolite levels to discriminate obese subjects with IS. We present for the first time a panel of metabolites comprising phospholipids to identify accurately the IS obesity metabotype and potentially MHO subjects. This model might also enable specialists to monitor the progression from IS to high IR in the obese population. Nevertheless, to that end, additional research needs to be carried out in a greater and independent cohort to validate this biomarker model in adipose tissue and identify it in a more accessible biological sample such as blood samples. Moreover, the progression of these lipid species will need to be explored in the pathological state, i.e., obesity with one or more comorbidities, to define normality intervals and a disease cutoff.

## Conclusions

In conclusion, our study has demonstrated the association of obesity with an important alteration in the composition of VAT. Obese subjects with IS present an alteration of the phospholipids containing C18-fatty acyl groups metabolism. This lipid remodeling might promote proinflammatory responses in VAT, which will be improved if the patient presents both conditions at the same time. The particular combination of GPE (18:2), GPI (18:0), GPS (18:0), GPC (18:0/18:1), GPC (18:2/18:2), and GPC (18:2/18:3) configures a sensitive and specific biomarker to distinguish subjects with an IS obesity metabotype from those with high IR obesity. This research was supported by Project PI13/01172, AC19/00096, CIBERFES, CIBEROBN, funded by Instituto de Salud Carlos III and co-funded by European Regional Development Fund; A way to make Europe. PI-0557-2013, co-funded by Fundación Progreso y Salud, 563 Consejería de Salud y Bienestar Social, Junta de Andalucía. CAL awarded by ICREA Academia 2018 and 2017SGR1546 grant from the Generalitat de Catalunya’s Agency AGAUR. M.P.-R. acknowledges the APIF fellowship [INSA-UB], A.M.-R. and S.T. acknowledge the Juan de la Cierva fellowship [MINECO].

## Supporting Information Available

Stratification of the groups and multimetabolite biomarker (Figure S1); statistical significance of discriminative metabolites according to the patient group in obesity, high IR, interaction of both metabolic statuses and discordant phenotypes of both (Table S1); enrichment analysis of the metabolic pathways altered in obesity (Table S2); and most discriminative metabolites in VAT in obesity obtained from multivariate RF analysis (Table S3) (PDF)

## Abbreviations

BMI: body mass index
CI: confidence interval
C18: 18 carbons
ESI: electrospray ionization
FG: fasting glucose
HDL: high-density lipoprotein
HILIC: hydrophilic interaction liquid chromatography
HOMA-IR: homeostatic model assessment-insulin resistance
IR: insulin resistance
IS: insulin sensitivity
*k*-NN: *k*-nearest neighbors algorithm
LASSO: least absolute shrinkage and selection operator
MHO: metabolically healthy obesity
MS/MS: tandem mass spectrometry
PUFA: polyunsaturated fatty acid
QC: quality control
GPC: glycerophosphatidylcholine
GPE: glycerophosphatidylethanolamine
GPI: glycerophosphatidylinositol
GPS: glycerophosphatidylserine
RF: random forest
RP-UPLC: reverse-phase ultraperformance liquid chromatography
ROC: receiver operating characteristic
SAT: subcutaneous adipose tissue
VAT: visceral adipose tissue
VIP: variable importance in projection
