# Supplementary material for: Visceral Adipose Tissue Phospholipid Signature of Insulin Sensitivity and Obesity
Source: J Proteome Res. 2021 Mar 24;20(5):2410–9. doi: 10.1021/acs.jproteome.0c00918 (PMC8631729; doi:10.1021/acs.jproteome.0c00918)
Supplement: Supplementary file 1 — pr0c00918_si_001.pdf [file pr0c00918_si_001.pdf]

Supporting Information File

Visceral adipose tissue phospholipid signature of insulin sensitivity and obesity.

Magalí Palau-Rodriguez[a,b†], Anna Marco-Ramell[a,b†], Patricia Casas-Agustench[a,b], Sara Tulipani[a,c], Antonio Miñarro[b,d], Alex Sanchez-Pla[b,d,e], Mora Murri[c,f], Francisco J Tinahones[c,f*], Cristina Andres-Lacueva[a,b*]

[†] equal contribution

[a]Biomarkers and Nutrimetabolomics Laboratory, Department of Nutrition, Food Sciences and Gastronomy, XaRTA, INSA, Faculty of Pharmacy and Food Sciences, University of Barcelona, Barcelona 08028, Spain

[b]CIBER Fragilidad y Envejecimiento Saludable (CIBERfes), Instituto de Salud Carlos III, Barcelona 08028, Spain

[c] Department of Endocrinology and Nutrition, Instituto de Investigación Biomédica de Malaga (IBIMA), Virgen de la Victoria University Hospital, Málaga University, Malaga 2910, Spain

[d]Genetics, Microbiology and Statistics Department, Biology Faculty, University of Barcelona, Barcelona 08028, Spain

[e]Statistics and Bioinformatics Unit, Vall d'Hebron Institut de Recerca (VHIR), Barcelona 08035, Spain

[f]CIBER Fisiopatología de la Obesidad y Nutrición (CIBERobn), Instituto de Salud Carlos III, Madrid 28029, Spain

*Corresponding authors:

Dr. Cristina Andres Lacueva. Biomarkers and Nutrimetabolomics Laboratory, Department of Nutrition, Food Sciences and Gastronomy, Faculty of Pharmacy and Food Sciences, University of Barcelona

Av. Joan XXIII, 27-31, 08028, Barcelona, Spain.

Phone: +34.934034840; Fax: +34.93403593.

Email address: candres@ub.edu

Dr. Francisco J Tinahones. Department of Endocrinology and Nutrition, Instituto de Investigación Biomédica de Malaga (IBIMA), Virgen de la Victoria University Hospital, Málaga University, 2910, Malaga, Spain.

Phone: +34.951932734

Email address: fjtinahones@uma.es

Supporting information

Supplemental Figure S1. Stratification of the groups and multimetabolite biomarker.

Supplemental Table S1. Statistical significance of discriminative metabolites according to the patient group in obesity, high IR, interaction of both metabolic statuses and discordant phenotypes of both.

Supplemental Table S2. Enrichment analysis of the metabolic pathways altered in obesity.

Supplemental Table S3. The most discriminative metabolites in VAT in obesity obtained from multivariate RF analysis.

Supplemental Figure S1. Stratification of the groups and multimetabolite biomarker.

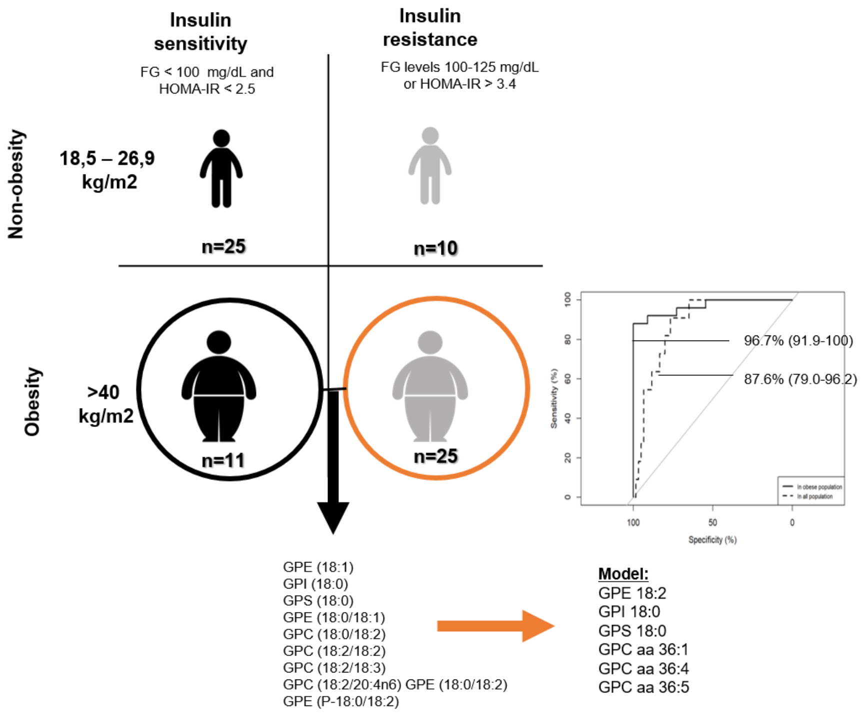

Supplemental Table S1. Statistical significance of discriminative metabolites according to the patient group in obesity, high IR, interaction of both metabolic statuses and discordant phenotypes of both. *P* values were corrected for multiple testing by the false discovery rate (adjusted *p* value) are. Metabolites are sorted alphabetically, according to their metabolic pathway. Abbreviations: IR, insulin resistance; IS, insulin sensitivity; OB, obesity; n.s., not significant. *Compounds identified with a very high level of confidence, but not validated with a standard; # *p* value <0.01 (raw *p* value).

		Adjusted *p* value					
**Metabolites**	**Metabolic pathway**	**OB**	**High IR**	**OB x high IR**	**OB, IS vs IR**	**high IR, Non-OB vs OB**	**Non-OB, IS vs IR**
Amino acids							
N-Acetylalanine	Alanine and Aspartate Metabolism	0.036	n.s.	n.s.	n.s.	n.s.	n.s.
N-Acetylasparagine	Alanine and Aspartate Metabolism	0.045	n.s.	n.s.	n.s.	n.s.	n.s.
Creatinine	Creatine Metabolism	0.001	n.s.	n.s.	n.s.	n.s.	n.s.
Guanidinoacetate	Creatine Metabolism	0.001	n.s.	n.s.	n.s.	n.s	n.s.
Beta-Citrylglutamate	Glutamate Metabolism	0.014	n.s.	n.s.	n.s.	0.034	n.s.
Glutamine	Glutamate Metabolism	0.013	n.s.	n.s.	n.s.	n.s.	n.s.
N-Acetylglutamate	Glutamate Metabolism	1.92E-06	n.s.	n.s.	n.s.	0.003	n.s.
N-Acetylglutamine	Glutamate Metabolism	0.002	n.s.	n.s.	n.s.	0.018	n.s.
2-Hydroxybutyrate/2-Hydroxyisobutyrate	Glutathione Metabolism	2.34E-05	n.s.	n.s.	n.s.	0.015	n.s.
5-Oxoproline	Glutathione Metabolism	0.007	n.s.	n.s.	n.s.	n.s.	n.s.
Cysteine-Glutathione Disulfide	Glutathione Metabolism	7.79E-04	n.s.	n.s.	n.s.	0.039	n.s.
Glutathione, Reduced (GSH)	Glutathione Metabolism	0.017	n.s.	n.s.	n.s.	n.s.	n.s.
Ophthalmate	Glutathione Metabolism	0.004	n.s.	n.s.	n.s.	n.s.	n.s.
Betaine	Glycine, Serine and Threonine Metabolism	9.52E-04	n.s.	n.s.	n.s.	n.s.	n.s.
Dimethylglycine	Glycine, Serine and Threonine Metabolism	0.006	n.s.	n.s.	n.s.	n.s.	n.s.
Glycine	Glycine, Serine and Threonine Metabolism	0.037	n.s.	n.s.	n.s.	n.s.	n.s.
N-Acetylglycine	Glycine, Serine and Threonine Metabolism	0.008	n.s.	n.s.	n.s.	n.s	n.s.

N-Acetylthreonine	Glycine, Serine and Threonine Metabolism	0.002	n.s.	n.s.	n.s.	n.s	n.s.
1-Methylhistidine	Histidine Metabolism	7.79E-04	n.s.	n.s.	n.s.	0.018	n.s.
1-Methylimidazoleacetate	Histidine Metabolism	0.002	n.s.	n.s.	n.s.	n.s.	n.s.
Histidine	Histidine Metabolism	0.010	n.s.	n.s.	n.s.	n.s.	n.s.
Imidazole Lactate	Histidine Metabolism	0.004	n.s.	n.s.	n.s.	n.s.	n.s.
2-Methylbutyrylcarnitine (C5)	Leucine, Isoleucine and Valine Metabolism	0.001	n.s.	n.s.	n.s.	0.045	n.s.
3-Methyl-2-Oxobutyrate	Leucine, Isoleucine and Valine Metabolism	0.026	n.s.	n.s.	n.s.	n.s	n.s.
3-Methyl-2-Oxovalerate	Leucine, Isoleucine and Valine Metabolism	0.023	n.s.	n.s.	n.s.	n.s.	n.s.
4-Methyl-2-Oxopentanoate	Leucine, Isoleucine and Valine Metabolism	0.019	n.s.	n.s.	n.s.	n.s.	n.s.
Alpha-Hydroxyisovalerate	Leucine, Isoleucine and Valine Metabolism	2.74E-04	n.s.	n.s.	n.s.	0.018	n.s.
Beta-Hydroxyisovalerate	Leucine, Isoleucine and Valine Metabolism	0.005	n.s.	n.s.	n.s.	n.s	n.s.
Isobutyrylcarnitine (C4)	Leucine, Isoleucine and Valine Metabolism	3.04E-04	n.s.	n.s.	n.s.	0.048	n.s.
Isoleucine	Leucine, Isoleucine and Valine Metabolism	0.034	n.s.	n.s.	n.s.	n.s.	n.s.
Isovalerylcarnitine (C5)	Leucine, Isoleucine and Valine Metabolism	1.31E-04	n.s.	n.s.	n.s.	n.s	n.s.
2-Aminoadipate	Lysine Metabolism	0.021	n.s.	n.s.	n.s.	n.s	n.s.
Pipecolate	Lysine Metabolism	0.019	n.s.	n.s.	n.s.	n.s.	n.s.
Cysteine	Methionine, Cysteine, SAM and Taurine Metabolism	0.002	n.s.	n.s.	n.s.	0.015	n.s.
S-Adenosylhomocysteine (SAH)	Methionine, Cysteine, SAM and Taurine Metabolism	0.037	n.s.	n.s.	n.s.	n.s.	n.s.
S-Methylcysteine	Methionine, Cysteine, SAM and Taurine Metabolism	0.002	n.s.	n.s.	n.s.	n.s	n.s.
Taurine	Methionine, Cysteine, SAM and Taurine Metabolism	0.004	n.s.	n.s.	n.s.	n.s	n.s.
Kynurenine	Tryptophan Metabolism	0.003	n.s.	n.s.	n.s.	n.s.	n.s.
Tryptophan Betaine	Tryptophan Metabolism	0.004	n.s.	n.s.	n.s.	n.s.	n.s.
Citrulline	Urea cycle, Arginine and Proline Metabolism	0.034	n.s.	n.s.	n.s.	n.s.	n.s.
Ornithine	Urea cycle, Arginine and Proline Metabolism	0.010	n.s.	n.s.	n.s.	n.s.	n.s.
Proline	Urea cycle, Arginine and Proline Metabolism	0.038	n.s.	n.s.	n.s.	n.s.	n.s.
Trans-4-Hydroxyproline	Urea cycle, Arginine and Proline Metabolism	0.045	n.s.	n.s.	n.s.	n.s.	n.s.
Urea	Urea cycle, Arginine and Proline Metabolism	5.18E-05	n.s.	n.s.	n.s.	0.018	n.s.
Carbohydrates							
Erythronate*	Aminosugar Metabolism	0.001	n.s.	n.s.	n.s.	n.s	n.s.
N-Acetylglucosamine/N-	Aminosugar Metabolism	0.011	n.s.	n.s.	n.s.	n.s.	n.s.

Acetylgalactosamine							
N-Acetylglucosaminylasparagine	Aminosugar Metabolism	0.045	n.s.	n.s.	n.s.	n.s.	n.s.
Mannose	Fructose, Mannose and Galactose Metabolism	3.94E-04	n.s.	n.s.	n.s.	0.018	n.s.
1,5-Anhydroglucitol (1,5-AG)	Glycolysis, Gluconeogenesis, and Pyruvate Metabolism	0.003	n.s.	n.s.	n.s.	n.s.	n.s.
3-Phosphoglycerate	Glycolysis, Gluconeogenesis, and Pyruvate Metabolism	0.003	n.s.	n.s.	n.s.	0.038	n.s.
Glucose	Glycolysis, Gluconeogenesis, and Pyruvate Metabolism	0.027	n.s.	n.s.	n.s.	n.s.	n.s.
Glycerate	Glycolysis, Gluconeogenesis, and Pyruvate Metabolism	0.001	n.s.	n.s.	n.s.	0.045	n.s.
Lactate	Glycolysis, Gluconeogenesis, and Pyruvate Metabolism	1.57E-05	n.s.	n.s.	n.s.	0.005	n.s.
Phosphoenolpyruvate (PEP)	Glycolysis, Gluconeogenesis, and Pyruvate Metabolism	0.018	n.s.	n.s.	n.s.	n.s	n.s.
Cofactors and vitamins							
Threonate	Ascorbate and Aldarate Metabolism	0.001	n.s.	n.s.	n.s.	0.043	n.s.
Heme	Hemoglobin and Porphyrin Metabolism	0.010	n.s.	n.s.	n.s.	0.007	n.s.
N1-Methyl-2-Pyridone-5-Carboxamide	Nicotinate and Nicotinamide Metabolism	0.026	n.s.	n.s.	n.s.	n.s.	n.s.
Alpha-Tocopherol	Tocopherol Metabolism	0.002	n.s.	n.s.	n.s.	n.s.	n.s.
Gamma-Tocopherol/Beta-Tocopherol	Tocopherol Metabolism	0.045	n.s.	n.s.	n.s.	n.s.	n.s.
Threonate	Ascorbate and Aldarate Metabolism	0.001	n.s.	n.s.	n.s.	n.s.	n.s.
Energy							
Alpha-Ketoglutarate	TCA Cycle	1.31E-04	n.s.	n.s.	n.s.	0.016	n.s.
Fumarate	TCA Cycle	0.013	n.s.	n.s.	n.s.	n.s.	n.s.
Malate	TCA Cycle	0.011	n.s.	n.s.	n.s.	n.s.	n.s.
Succinate	TCA Cycle	1.31E-04	n.s.	n.s.	n.s.	0.016	n.s.
Succinylcarnitine (C4-DC)	TCA Cycle	0.045	n.s.	n.s.	n.s.	n.s.	n.s.
Lipids							
Deoxycarnitine	Carnitine Metabolism	0.038	n.s.	n.s.	n.s.	n.s.	n.s.
Ceramide (d18:1/14:0, d16:1/16:0)*	Ceramides	0.021	n.s.	n.s.	n.s.	n.s	n.s.
Stearoyl Ethanolamide	Endocannabinoid	0.004	n.s.	n.s.	n.s.	n.s.	n.s.

Propionylcarnitine (C3)	Fatty Acid Metabolism (also BCAA Metabolism)	0.026	n.s.	n.s.	n.s.	n.s.	n.s.
3-Hydroxybutyrylcarnitine (1)	Fatty Acid Metabolism(Acyl Carnitine)	0.013	n.s.	n.s.	n.s.	n.s.	n.s.
Acetylcarnitine (C2)	Fatty Acid Metabolism(Acyl Carnitine)	0.010	n.s.	n.s.	n.s.	n.s	n.s.
Linoleoylcarnitine (C18:2)*	Fatty Acid Metabolism(Acyl Carnitine)	0.038	n.s.	n.s.	n.s.	n.s.	n.s.
Myristoleoylcarnitine (C14:1)*	Fatty Acid Metabolism(Acyl Carnitine)	n.s.	n.s.	n.s.	n.s.	0.046	n.s.
Oleoylcarnitine (C18:1)	Fatty Acid Metabolism(Acyl Carnitine)	0.009	n.s.	n.s.	n.s.	n.s	n.s.
Palmitoleoylcarnitine (C16:1)*	Fatty Acid Metabolism(Acyl Carnitine)	n.s.	n.s.	n.s.	n.s.	0.044	n.s.
Palmitoylcarnitine (C16)	Fatty Acid Metabolism(Acyl Carnitine)	0.002	n.s.	n.s.	n.s.	0.041	n.s.
2-Hydroxyglutarate	Fatty Acid, Dicarboxylate	3.52E-04	n.s.	n.s.	n.s.	0.016	n.s.
Glycerol	Glycerolipid Metabolism	6.75E-04	n.s.	n.s.	n.s.	0.015	n.s.
Glycerol 3-Phosphate	Glycerolipid Metabolism	0.003	n.s.	n.s.	n.s.	n.s.	n.s.
Glycerophosphoglycerol	Glycerolipid Metabolism	0.032	n.s.	n.s.	n.s.	n.s.	n.s.
3-Hydroxybutyrate (BHBA)	Ketone Bodies	0.024	n.s.	n.s.	n.s.	n.s.	n.s.
1-Linoleoyl-GPE (18:2)*	Lysolipid	0.035	n.s.	n.s.	0.017	0.026	n.s.
1-Oleoyl-GPE (18:1)	Lysolipid	n.s.	n.s.	n.s.	n.s.#	n.s.	n.s.
1-Stearoyl-GPI (18:0)	Lysolipid	n.s.	n.s.	n.s.	n.s.#	n.s.	n.s.
1-Stearoyl-GPS (18:0)	Lysolipid	n.s.	n.s.	n.s.	n.s.#	n.s.	n.s.
1-(1-Enyl-Oleoyl)-2-Linoleoyl-GPE (P-18:1/18:2)*	Lysoplasmalogen	2.74E-04	n.s.	n.s.	n.s.	0.005	n.s.
1,2-Dilinoleoyl-GPC (18:2/18:2)	Phospholipid Metabolism	n.s.	n.s.	n.s.	n.s.#	0.045	n.s.
1-Linoleoyl-2-Arachidonoyl-GPC (18:2/20:4n6)*	Phospholipid Metabolism	n.s.	n.s.	n.s.	n.s	0.045	n.s.
1-Linoleoyl-2-Linolenoyl-GPC (18:2/18:3)*	Phospholipid Metabolism	n.s.	n.s.	n.s.	n.s.#	0.043	n.s.
1-Oleoyl-2-Linoleoyl-GPC (18:1/18:2)*	Phospholipid Metabolism	0.019	n.s.	n.s.	n.s.	n.s	n.s.
1-Stearoyl-2-Linoleoyl-GPC (18:0/18:2)	Phospholipid Metabolism	n.s.	n.s.	n.s.	n.s.#	n.s.	n.s.
1-Stearoyl-2-Linoleoyl-GPE (18:0/18:2)	Phospholipid Metabolism	n.s.	n.s.	n.s.	n.s.#	n.s.	n.s.
1-Stearoyl-2-Oleoyl-GPC (18:0/18:1)	Phospholipid Metabolism	0.036	n.s.	n.s.	n.s.#	n.s	n.s.
Choline	Phospholipid Metabolism	9.60E-04	n.s.	n.s.	n.s.	0.045	n.s.
Glycerophosphoserine*	Phospholipid Metabolism	0.014	n.s.	n.s.	n.s.	n.s.	n.s.

1-(1-Enyl-Palmitoyl)-2-Linoleoyl-GPE (P-16:0/18:2)*	Plasmalogen	0.030	n.s.	n.s.	n.s.	n.s	n.s.
1-(1-Enyl-Palmitoyl)-2-Palmitoleoyl-GPC (P-16:0/16:1)*	Plasmalogen	0.026	n.s.	n.s.	n.s.	n.s.	n.s.
1-(1-Enyl-Stearoyl)-2-Linoleoyl-GPE (P-18:0/18:2)*	Plasmalogen	3.52E-04	n.s.#	n.s.	n.s.#	0.045	n.s.
1-(1-Enyl-Stearoyl)-2-Oleoyl-GPE (P-18:0/18:1)	Plasmalogen	0.005	n.s.	n.s.	n.s.	n.s	n.s.
Arachidonate (20:4n6)	Polyunsaturated Fatty Acid (n3 and n6)	0.008	n.s.	n.s.	n.s.	n.s.	n.s.
Docosahexaenoate (DHA, 22:6n3)	Polyunsaturated Fatty Acid (n3 and n6)	0.013	n.s.	n.s.	n.s.	n.s.	n.s.
Docosapentaenoate (DPA, 22:5n3)	Polyunsaturated Fatty Acid (n3 and n6)	0.008	n.s.	n.s.	n.s.	0.016	n.s.
Docosapentaenoate (DPA, 22:5n6)	Polyunsaturated Fatty Acid (n3 and n6)	n.s.	n.s.	n.s.	n.s.	0.003	n.s.
Glycosyl-N-Palmitoyl-Sphingosine (d18:1/16:0)	Sphingolipid Metabolism	0.034	n.s.	n.s.	n.s.	0.047	n.s.
Lactosyl-N-Nervonoyl-Sphingosine (d18:1/24:1)*	Sphingolipid Metabolism	0.043	n.s.	n.s.	n.s.	n.s.	n.s.
N-Nervonoyl-Hexadecasphingosine (d16:1/24:1)*	Sphingolipid Metabolism	0.020	n.s.	n.s.	n.s.	n.s	n.s.
N-Nervonoyl-Sphingadiene (d18:2/24:1)*	Sphingolipid Metabolism	1.21E-04	n.s.	n.s.	n.s.	0.045	n.s.
N-Palmitoyl-Sphingadienine (d18:2/16:0)*	Sphingolipid Metabolism	7.08E-04	n.s.	n.s.	n.s.	0.045	n.s.
N-Stearoyl-Sphingadienine (d18:2/18:0)*	Sphingolipid Metabolism	0.001	n.s.	n.s.	n.s.	n.s	n.s.
Sphingomyelin (d17:2/16:0, d18:2/15:0)*	Sphingolipid Metabolism	0.036	n.s.	n.s.	n.s.	n.s.	n.s.
Sphingomyelin (d18:1/18:1, d18:2/18:0)	Sphingolipid Metabolism	0.004	n.s.	n.s.	n.s.	n.s	n.s.
Sphingomyelin (d18:1/20:1, d18:2/20:0)*	Sphingolipid Metabolism	0.038	n.s.	n.s.	n.s.	n.s.	n.s.
Sphingomyelin (d18:1/22:2, d18:2/22:1, d16:1/24:2)*	Sphingolipid Metabolism	0.002	n.s.	n.s.	n.s.	0.043	n.s.
Sphingomyelin (d18:2/14:0, d18:1/14:1)*	Sphingolipid Metabolism	0.044	n.s.	n.s.	n.s.	n.s.	n.s.
Sphingomyelin (d18:2/16:0, d18:1/16:1)*	Sphingolipid Metabolism	0.025	n.s.	n.s.	n.s.	n.s	n.s.

Sphingomyelin (d18:2/24:1, d18:1/24:2)*	Sphingolipid Metabolism	0.026	n.s.	n.s.	n.s.	n.s.	n.s.
Sphingomyelin (d18:2/24:2)*	Sphingolipid Metabolism	0.001	n.s.	n.s.	n.s.	n.s.	n.s.
4-Cholesten-3-One	Sterol	0.011	n.s.	n.s.	n.s.	n.s.	n.s.
<u>Nucleotides</u>							
Hypoxanthine	Purine Metabolism, (Hypo)Xanthine/Inosine containing	0.024	n.s.	n.s.	n.s.	n.s.	n.s.
Urate	Purine Metabolism, (Hypo)Xanthine/Inosine containing	3.24E-05	n.s.	n.s.	n.s.	0.018	n.s.
Xanthine	Purine Metabolism, (Hypo)Xanthine/Inosine containing	9.52E-04	n.s.	n.s.	n.s.	n.s.	n.s.
7-Methylguanine	Purine Metabolism, Guanine containing	0.004	n.s.	n.s.	n.s.	0.003	n.s.
3-Ureidopropionate	Pyrimidine Metabolism, Uracil containing	0.003	n.s.	n.s.	n.s.	0.039	n.s.
Pseudouridine	Pyrimidine Metabolism, Uracil containing	2.34E-05	n.s.	n.s.	n.s.	0.003	n.s.
Uracil	Pyrimidine Metabolism, Uracil containing	1.24E-04	n.s.	n.s.	n.s.	0.005	n.s.
<u>Peptides</u>							
Glycylleucine	Dipeptide	0.038	n.s.	n.s.	n.s.	n.s.	n.s.
Phenylalanylglycine	Dipeptide	6.75E-04	n.s.	n.s.	n.s.	0.003	n.s.
Gamma-Glutamylglutamine	Gamma-glutamyl Amino Acid	8.62E-05	n.s.	n.s.	n.s.	0.018	n.s.
Gamma-Glutamylthreonine	Gamma-glutamyl Amino Acid	3.94E-04	n.s.	n.s.	n.s.	0.004	n.s.

Supplemental Table S2. Enrichment analysis of the metabolic pathways altered in obesity. Enrichment and p-value mirror the magnitude of the alteration of these pathways. Metabolic pathways are sorted according to the p-value of the alteration.

Metabolic pathway	Significant	Detected	Ratio S/D[a]	Ratio All S/D[b]	Enrichment	p-value[c]
**OBESITY**						
Leucine, isoleucine and valine metabolism	9	12	0.75	0.28	2.68	0.001
Glutathione metabolism	5	6	0.83	0.28	2.98	0.007
TCA cycle	5	6	0.83	0.28	2.98	0.007
Glycolysis, gluconeogenesis and pyruvate metabolism	6	9	0.67	0.28	2.38	0.017
Glycerolipid metabolism	3	3	1.00	0.28	3.58	0.021
Glycine, serine and threonine metabolism	5	8	0.63	0.28	2.24	0.042

a) Ratio significant metabolites / detected metabolites in a particular metabolic pathway
b) Ratio all significant metabolites (118) / all detected metabolite (422)
c) Calculated with hypergeometric test

Supplemental Table S3. The most discriminative metabolites in VAT in obesity obtained from multivariate RF analysis. The smaller the VIP value is, the more discriminant the metabolite is. Metabolites are sorted according to their VIP value. Abbreviations: aa, acyl-acyl; ae, acyl-alkyl; PC, phosphatidylcholine; PE, phosphatidylethanolamine; VIP, variable importance in projection.

**VIP**	**Name**	**Metabolic pathway**	**Chemical class**
11.47	Urate	Purine Metabolism, (Hypo)Xanthine/Inosine containing	Nucleotide
12.45	Lactate	Glycolysis, Gluconeogenesis, and Pyruvate Metabolism	Carbohydrate
16.50	N-Acetylglutamate	Glutamate Metabolism	Amino acid
18.47	Urea	Urea cycle, Arginine and Proline Metabolism	Amino acid
25.02	2-Hydroxybutyrate/2-Hydroxyisobutyrate	Glutathione Metabolism	Amino acid
25.37	Succinate	TCA Cycle	Energy
27.49	1-(1-Enyl-Stearoyl)-2-Oleoyl-GPE (GPE ae 18:0/18:1,GPE ae 36:1)	Plasmalogen	Lipid
27.91	1-(1-Enyl-Stearoyl)-2-Linoleoyl-GPE (GPE ae 18:0/18:2, GPE ae 36:2)	Plasmalogen	Lipid
35.45	Alpha-Hydroxyisovalerate	Leucine, Isoleucine and Valine Metabolism	Amino acid
36.17	Gamma-Glutamylglutamine	Gamma-glutamyl Amino acid	Peptide
38.09	Pseudouridine	Pyrimidine Metabolism, Uracil containing	Nucleotide
39.63	N-Nervonoyl-Sphingadiene (d18:2/24:1)*	Sphingolipid Metabolism	Lipid
40.93	Uracil	Pyrimidine Metabolism, Uracil containing	Nucleotide
42.88	Erythronate*	Aminosugar Metabolism	Carbohydrate
49.20	1-(1-Enyl-Oleoyl)-2-Linoleoyl-GPE* (GPE ae 18:1/18:2, GPE ae 36:3)	Lysoplasmalogen	Lipid
49.95	Glycerol	Glycerolipid Metabolism	Lipid
52.96	Isobutyrylcarnitine (C4)	Leucine, Isoleucine and Valine Metabolism	Amino acid
53.49	1-Oleoyl-2-Linoleoyl-PC* (GPC aa 18:1/18:2, GPC aa 36:3)	Phospholipid Metabolism	Lipid

62.09	Choline	Phospholipid Metabolism	Lipid
66.45	7-Methylguanine	Purine Metabolism, Guanine containing	Nucleotide
67.48	Fumarate	TCA Cycle	Energy
67.71	Stearoyl Ethanolamide	Endocannabinoid	Lipid
73.92	S-Methylcysteine	Methionine, Cysteine, SAM and Taurine Metabolism	Amino acid
74.33	Alpha-Ketoglutarate	TCA Cycle	Energy
76.67	Glycerate	Glycolysis, Gluconeogenesis, and Pyruvate Metabolism	Carbohydrate
77.67	Glycerol 3-Phosphate	Glycerolipid Metabolism	Lipid
79.10	Isovalerylcarnitine (C5)	Leucine, Isoleucine and Valine Metabolism	Amino acid
79.78	Beta-Citrylglutamate	Glutamate Metabolism	Amino acid
81.65	5-Oxoproline	Glutathione Metabolism	Amino acid
82.29	Mannose	Fructose, Mannose and Galactose Metabolism	Carbohydrate
82.61	Guanidinoacetate	Creatine Metabolism	Amino acid
82.64	1-Methylhistidine	Histidine Metabolism	Amino acid
